# Appendicitis and COVID: cause or effect?

**DOI:** 10.1186/s42269-022-00798-w

**Published:** 2022-04-21

**Authors:** Abdus Salam Raju, Aditya Thomas Benjamin, Tristan Rutland, Luke Liu, Paul Lambrakis

**Affiliations:** 1 Acute Care Surgery Unit, Liverpool Hospital, 75 Elizabeth Street, Liverpool, NSW 2170 Australia; 2 School of Medicine, Western Sydney University, Sydney, NSW Australia; 3 Department of Anatomical Pathology, NSW Health Pathology, Liverpool Hospital, Liverpool, NSW Australia; 4 School of Medicine, University of New South Wales, Sydney, NSW Australia

**Keywords:** COVID-19 infection, Appendicitis, Veno-occlusive disease, Veno-occlusive vasculitis

## Abstract

**Background:**

Vasculitis and phlebitis with vascular occlusion within appendix specimen is rare. Several authors have reported COVID-19 related veno-occlusive disease in hepatic pathology, but lymphoid aggregation with phlebitis is unusual in appendix specimen. We present a case with medium size venule phlebitis in an appendix of a patient recovered from COVID-19 infection.

**Case presentation:**

A 27-year-old who recently recovered from COVID-19 infection 12 weeks prior, presented with acute appendicitis, confirmed on computed tomography and operative findings. He underwent an uneventful laparoscopic appendicectomy. Histopathology showed veno-occlusive vasculitis within the appendix specimen.

**Conclusions:**

Veno-occlusive disease within the appendix is uncommon. Emerging data suggest COVID-19 infection can cause systemic vascular complications. We herein report a case of healthy patient with no past medical history with an unusual findings of medium vessels phlebitis within the appendix post COVID-19 infection.

## Background

Lymphocytic phlebitis is a rare finding in the appendix and is usually associated with systemic causes. There has been increasing evidence around the role that endothelial injury plays in the COVID-19 disease process and a recent article (Lagana et al. 2020) describes the presence of phlebosclerosis which was reminiscent of veno-occlusive disease. According to the most commonly believed theory, luminal obstruction leads inflammation of the appendix. As a result, ischemia occurs, and if untreated inflamed appendix eventually perforates.

A case report of a child with Multisystem Inflammatory Syndrome, presenting with features of acute appendicitis, have shown acute vasculitis of mesenteric arteries and veins in the resected appendix (Jackson et al. 2020). Another case report in a 59-year-old male with COVID-19 showed acute vasculitis of the gallbladder with obliteration of the vessels (Bruni et al. 2020).

Other cases of unusual complications after COVID-19 infection may theoretically also be related to small vessel inflammation and thrombosis (Becker 2020). Giant Coronary Artery Aneurysm was temporarily associated with COVID-19 infection (Richardson et al. 2021). A literature review (Jedlowski and Jedlowski 2021) showed an association between Immunoglobulin A vasculitis in COVID-19 patients and all cases were in male patients. There is also a high frequency of clinical characteristics such as skin, gastrointestinal and renal involvement respectively (Jedlowski and Jedlowski 2021).

In addition, there has been increasing concern regarding potential of thrombo-embolism in patients vaccinated for COVID-19, the process of which is yet to be determined (Oldenburg et al. 2021). Particular attentions may be needed to investigate potential host related genetic predispositions of an abnormal immune response to specific virus proteins and also the relationship between these factors and the activation of the coagulation cascade. If such a causal relationship can be identified, there may be a way to determine which host is susceptible to thromboembolic complications of COVID-19 and/or COVID-19 vaccination.

## Case presentation

A 27-year-old man presented with a 4-day history of non-migratory right lower quadrant pain to our emergency department. He was diagnosed with acute appendicitis with the help of computed tomography (CT) scan and he subsequently underwent a laparoscopic appendicectomy. His past history was significant in that he recovered from COVID-19 infection 2 months prior to presentation. The histopathological findings revealed features of lymphocytic type phlebitis, which is a rare finding. In this case, the histopathological features are reportedly similar to the features seen in veno-occlusive disease associated with liver disease related to COVID-19. Possible relationships between this patient’s COVID-19 infection and the unusual histology are considered.

Our patient did not have chronic abdominal pain symptoms or symptoms of inflammatory bowel disease. He had no history of autoimmune related disease symptoms. His background medical history included a previously investigated mild persisting neutropenia most likely a benign ethnic neutropenia.

He had both ultrasound and computed tomography scan imaging performed which confirmed appendicitis. Initial full blood count revealed a white cell count (WBC) of 3.8 × 10^9^/L with predominantly a neutropenia of 1.4 × 10^9^/L consistent with his known haematological history. He had a minimally elevated C-reactive protein (CRP) of 7 mg/L. All other blood parameters were normal.

We performed a laparoscopic appendicectomy on the same day as his presentation to our hospital. At the time of laparoscopy. We noted an abnormally dilated (75 mm in length and 15 mm diameter) and inflamed appendix with reactive peritoneal free fluid. There were no other unusual findings on laparoscopic assessment. Cytology of the peritoneal fluid revealed mesothelial cells, macrophages and lymphocytes. Our patient made an uneventful post-operative recovery.

Of incidental note, our patient had been diagnosed with COVID-19 2 months prior to this presentation. The diagnosis had been made on SARS-Cov2-RNA nasal and throat swab during an episode of flu-like symptoms. He was managed with community isolation and was predominantly asymptomatic for the duration of his isolation. He had remained asymptomatic in the interim period after clearance from the Public Health unit.

The histology showed features of an interval (chronic) appendicitis with transmural fibrosis with some areas of organisation. Of significant interest, there were focal areas of a lymphocytic phlebitis with some occlusion of the vessels. Elastic stains (EVG) highlighted the occluded vessels. Immunohistochemistry revealed only scattered plasma cells and there was no increase in IgG4 plasma cells. We were mindful of the fact that our patient was not diagnosed with a thrombus elsewhere.

Microscopic analysis showed areas of mucosal ulceration and architectural distortion of the appendiceal glands. There were areas of scattered large lymphoid aggregated extending to the mesoappendix with germinal centres. Patchy area of phlebitis was seen on EVG stain. Although the arterial structures were unremarkable, medium sized venules showed evidence of phlebitis. Immunohistochemistry for CD138 showed scattered plasma cells only on IgG 4 (numerous IgG positive cells).

## Conclusions

To our knowledge, this is the first reported case of a lymphocytic phlebitis with thrombosis occurring in this setting. Given the unusual nature of our case’s histopathological findings and the association with resolved COVID-19 infection, we postulate a possible causative relationship. The longer term systemic effects of COVID-19 are not yet understood and our knowledge of possible microvascular complications is developing. The exact link between virus related factors or specific virus proteins and the host immune response needs to be further investigated.

## Data Availability

Medical and histopathological data was retrieved from our hospital record office.

## Abbreviations

CT: Computed tomography
WBC: White blood cells
CRP: C reactive protein
EVG: Elastic stains
